# Supplementary material for: Empirical methods for controlling false positives and estimating confidence in ChIP-Seq peaks
Source: BMC Bioinformatics. 2008 Dec 5;9:523. doi: 10.1186/1471-2105-9-523 (PMC2628906; doi:10.1186/1471-2105-9-523)
Supplement: Additional File 1 — A variety of html documents from the USeq web site detailing the available applications, their best usage, output file type descriptions, command line menus, etc. [file 1471-2105-9-523-S1.zip › USeqUserGuides/JavaDoc.zip!/JavaDoc/util/bio/annotation/TransGroup.html]

TransGroup


|  |  |  |
| --- | --- | --- |
| **Overview** \| **Package** \| **Class** \| **Use** \| **Tree** \| **Deprecated** \| **Index** \| **Help** | |  |
| **PREV CLASS**   **NEXT CLASS** | **FRAMES**    **NO FRAMES**     **All Classes** |  |
| SUMMARY: NESTED \| FIELD \| CONSTR \| METHOD | DETAIL: FIELD \| CONSTR \| METHOD |  |


---


## util.bio.annotation Class TransGroup

```
java.lang.Object
  util.bio.annotation.TransGroup
```

**All Implemented Interfaces:**: java.io.Serializable

---

``` public class TransGroup extends java.lang.Object implements java.io.Serializable ```

A TransGroup describes a product derived from a GeneGroup contains one transcript, possibly a translation, and a possibly a set of exons.

**See Also:**: Serialized Form

---

**Constructor Summary**

`TransGroup(Transcript transcript,
Translation translation,
ExonIntron[] exons)`


| **Method Summary** | |
| --- | --- |
| `ExonIntron[]` | `getExons()` |
| `int[][]` | `getExtractedExons()` |
| `Transcript` | `getTranscript()` |
| `Translation` | `getTranslation()` |
| `java.lang.String` | `toString()` |

**Methods inherited from class java.lang.Object**

`equals, getClass, hashCode, notify, notifyAll, wait, wait, wait`

**Constructor Detail**

### TransGroup

```
public TransGroup(Transcript transcript,
                  Translation translation,
                  ExonIntron[] exons)
```


**Method Detail**

### toString

```
public java.lang.String toString()
```

:   **Overrides:**: `toString` in class `java.lang.Object`

---


### getExons

```
public ExonIntron[] getExons()
```

---


### getTranscript

```
public Transcript getTranscript()
```

---


### getTranslation

```
public Translation getTranslation()
```

---


### getExtractedExons

```
public int[][] getExtractedExons()
```


---


|  |  |  |
| --- | --- | --- |
| **Overview** \| **Package** \| **Class** \| **Use** \| **Tree** \| **Deprecated** \| **Index** \| **Help** | |  |
| **PREV CLASS**   **NEXT CLASS** | **FRAMES**    **NO FRAMES**     **All Classes** |  |
| SUMMARY: NESTED \| FIELD \| CONSTR \| METHOD | DETAIL: FIELD \| CONSTR \| METHOD |  |


---
